# The largest sauropodomorph skull from the Lower Jurassic Lufeng Formation of China

**DOI:** 10.7717/peerj.18629

**Published:** 2024-12-12

**Authors:** Qian-Nan Zhang, Lei Jia, Tao Wang, Yu-Guang Zhang, Hai-Lu You

**Affiliations:** 1 Key Laboratory of Vertebrate Evolution and Human Origins, Institute of Vertebrate Paleontology and Paleoanthropology, Chinese Academy of Sciences, Beijing, China; 2 State Key Laboratory of Palaeobiology and Stratigraphy, Nanjing Institute of Geology and Palaeontology, Chinese Academy of Sciences, Nanjing, China; 3 University of Chinese Academy of Sciences, Beijing, China; 4 ShanXi Museum of Geology, Taiyuan, China; 5 Dinosaur Fossil Conservation and Research Center, Bureau of Natural Resources of Lufeng County, Chuxiong, China; 6 National Natural History Museum of China, Beijing, China

**Keywords:** Sauropodomorpha, Sauropodiformes, Lufeng Formation, Lower Jurassic, Skull

## Abstract

The Lower Jurassic Lufeng Formation of China has long been recognized for its diverse early-diverging sauropodomorph dinosaurs, with eight genera and ten species, representing more than half the Laurasian records. In this paper, we describe a new genus and species of non-sauropodan sauropodomorph, *Lishulong wangi* gen. et sp. nov., from Yunnan Province in southwestern China. This new taxon is represented by a partial skeleton including the skull and nine articulated cervical vertebrae, which differs from other Lufeng forms in both cranial and cervical characteristics. It bears several autapomorphies of the nasal process, the maxillary neurovascular foramen, and the cervical neural spine. Phylogenetic analysis reveals that *Lishulong* is an early-diverging member of the Sauropodiformes, and the sister-taxon of *Yunnanosaurus*. Elucidating the novel osteology of *Lishulong*, it possessed the largest sauropodomorph cranial material currently identified from the Lufeng Formation, not only enriches the diversity of the Lufeng dinosaur assemblage, but also enhances our understanding of the character evolution in early-diverging sauropodiforms. Furthermore, information about paleobiogeographic distributions indicates that Early Jurassic sauropodomorphs, especially Chinese taxa, have maintained multiple dispersions and exchanges within Pangaea.

## Introduction

Non-sauropodan sauropodomorphs were the dominant group of herbivores from the Norian until the end of the Early Jurassic, when they were replaced by sauropods (Barrett & Upchurch, 2005; Mannion et al., 2011). Since *Thecodontosaurus* was first established (Riley & Stutchbury, 1836), over 40 valid genera of non-sauropodan sauropodomorphs have been reported worldwide (Müller, 2020). Most of these genera were identified from Gondwana, mainly recovered in South America and southern Africa (Langer et al., 1999). The first well-known Asian genus, *Lufengosaurus*, was described by Young (1941a). Since then, six other genera from the Lower Jurassic Lufeng Formation (LJLF) have been documented (Lü et al., 2007; Sekiya et al., 2013; Wang, You & Wang, 2017; Young, 1941b, 1942, 1947, 1951; Zhang et al., 2018; Zhang & Yang, 1995). The Early Jurassic epoch was a crucial period in tracking the early radiation and diversification of sauropodomorph dinosaurs. Nearly all the non-sauropodan sauropodomorphs currently recovered in China are reported from Yunnan Province, and the LJLF is the richest fossil-bearing Mesozoic unit in the province. Although these Lufeng materials are well preserved, and the abundance is high, they are under-represented in comparative studies and cladistic analyses, especially the rare skulls. Here, we report on *Lishulong wangi* gen. et sp. nov., from the LJLF of China, a new taxon that lies in the heart of the early sauropodomorph-sauropod transition. This new specimen, apart from possessing a combination of characters distinct from those of coeval dinosaurs, shows several unique characteristic traits of the skull and cervical vertebrae. *Lishulong* further increases the abundance of non-sauropodan sauropodomorphs, while providing more support for the Asian origin of Sauropodiformes, and indicating rapid radiation of this clade in southwestern China during the Early Jurassic epoch.

## MATERIAL and METHODS

**Figure 1:**
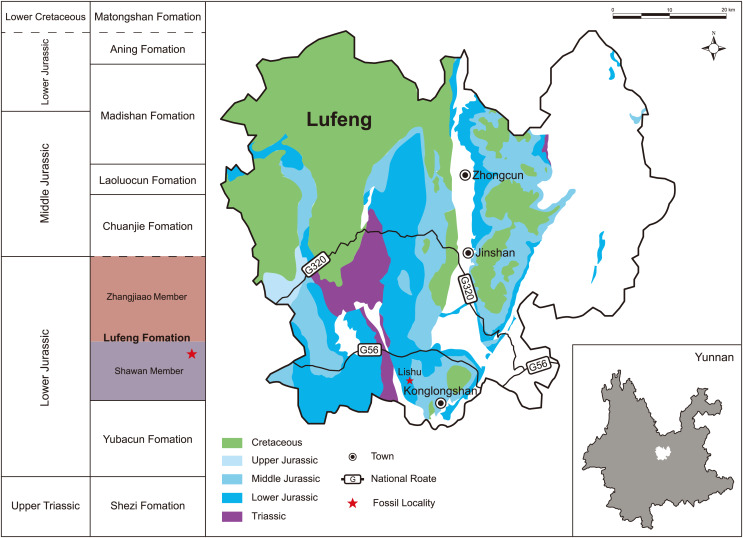
Geographic and stratigraphic distribution of the locality of *Lishulong wangi* gen. et sp. nov. The generalized stratigraphic section is modified from Fang et al. (2000).

The new specimen LFGT-ZLJ0011 was discovered and excavated by the staff of the Bureau of Natural Resources of Lufeng County from the Dalishu, Jiudu Village in Konglongshan Town (Fig. 1), which is very close to where the earliest Chinese theropod dinosaur, *Panguraptor lufengensis* (You et al., 2014), was found. The fossil locality is situated in the Lufeng Dinosaur National Geopark of Yunnan Province; the specimen is currently on display at the museum of Lufeng World Dinosaur Valley. All the bones were collected by plaster jackets, and then manually prepared using pin vices, brushes, and air micro grinders. Measurements of every isolated element were taken with a pair of sliding calipers for distances or diameters between 0 and 150 mm. For lengths greater than 150 mm and irregular surfaces, a measuring tape was employed. All photographs were captured by Mr. Wei Gao, the cameraman from IVPP using a Canon EOS 5D and relevant accessories.

The specimen is described in detail using standard comparative anatomical techniques. Well-preserved skulls are rare in sauropodomorphs; however, the Lufeng Formation yields abundant such cranial materials, such as *Lufengosaurus*, *Jingshanosaurus* and *Yizhousaurus*, which are also easy to be personally examined by the authors. Nonetheless, sauropodomorph taxa from other regions with skulls and cervical vertebrae have also been used for comparison, based on literature and specimen photographs shared by peers. These data are then used to establish possible autapomorphies of *Lishulong* and evaluate its cladistic position. A phylogenetic analysis was performed based on the data matrix (Zhang et al., 2018) that was comprised of 61 taxa and 364 characters, of which 120 regard craniodental homologies. We also accepted the revised cranial coding of *Jingshanosaurus* by Zhang et al. (2020). In the modified dataset, characters were equally weighted and the following multistate ones were treated as additive: 8, 13, 19, 23, 40, 57, 69, 92, 102, 117, 121, 131, 134, 145, 148, 150, 151, 158, 163, 168, 171, 178, 185, 208, 211, 218, 226, 231, 238, 246, 254, 257, 270, 282, 303, 309, 317, 337, 350, 353, 355, 360, 364. Scorings for the characters were managed in the software Mesquite v3.04 (Maddison & Maddison, 2011). The matrix was exported into TNT v1.1 (Goloboff, Farris & Nixon, 2008) for heuristic searches under the parsimony criterion. We searched for the optimal tree from 1,000 Wagner addition sequences, holding 10 of the shortest trees per replication and swapping topologies using tree bisection and reconnection. Bremer support and bootstrap resampling of 1,000 replications were also conducted in TNT.

The biogeographic analysis using the BioGeoBEARS package in R (Matzke, 2013, 2014) required a fully resolved topology of the phylogenetic tree. Therefore, the strict consensus tree obtained from cladistic analysis was selected along with priori pruning of four high-rank taxonomic units, namely Crurotarsi, Ornithischia, Neotheropoda and Neosauropoda, and *Glacialisaurus* found in Antarctica, which was not incorporated into the geographic ranges selected for biogeographic analysis. Log-likelihood ratio tests were performed and Akaike Information Criterion (AIC) values were calculated to identify which one of the six biogeographic models (Dispersal-Extinction-Cladogenesis (DEC), DEC + J, DIVALIKE, DIVALIKE + J, BAYAREALIKE, and BAYAREALIKE + J) in BioGeoBEARS had the maximum likelihood to yield available data. DEC and DIVALIKE models allow different forms of vicariance to occur at nodes, whereas BAYAREALIKE disallows vicariance and instead forces daughter lineages to inherit the range of their immediate ancestor (Matzke, 2013). Two sets of analyses were conducted adopting either ‘relaxed’ or ‘harsh’ versions of the dispersal multiplier matrix (Poropat et al., 2016; Xu et al., 2018). The matrix reflecting the interconnectivity of the eight continent units during the earliest time slice (251.9–227 Ma) for our stratified analysis is identical to the first time slice reported by Xu et al. (2018) because: (1) only a few taxa predate the Carnian age, which are not the foci of our analyses; (2) the arrangement of geographic units in our analysis has not changed much in this period, during which Pangaea had not started breaking apart (Smith, Smith & Funnell, 2004).

The electronic version of this article in Portable Document Format (PDF) will represent a published work according to the International Commission on Zoological Nomenclature (ICZN), and hence the new names contained in the electronic version are effectively published under that Code from the electronic edition alone. This published work and the nomenclatural acts it contains have been registered in ZooBank, the online registration system for the ICZN. The ZooBank LSIDs (Life Science Identifiers) can be resolved and the associated information viewed through any standard web browser by appending the LSID to the prefix http://zoobank.org/. The LSID for this publication is [urn:lsid:zoobank.org:pub:6A0C59EE-614C-462D-94C6-C28F86B3F9E7]. The online version of this work is archived and available from the following digital repositories: PeerJ, PubMed Central SCIE and CLOCKSS.

## Results

**Systematic Paleontology**

Dinosauria Owen, 1842

Saurischia Seeley, 1888

Sauropodomorpha von Huene, 1932 (*sensu* Sereno, 2007)

Massopoda Yates, 2007

Sauropodiformes Sereno, 2007 (*sensu* McPhee et al., 2014)

*Lishulong wangi* gen. et sp. nov.

Genus name [urn:lsid:zoobank.org:act:EE7FDF02-D7E8-422C-9853-62D7AD5271B2]

Species name [urn:lsid:zoobank.org:act:A1B589CE-0836-4048-B68E-3608F59FE574]

**Figure 2:**
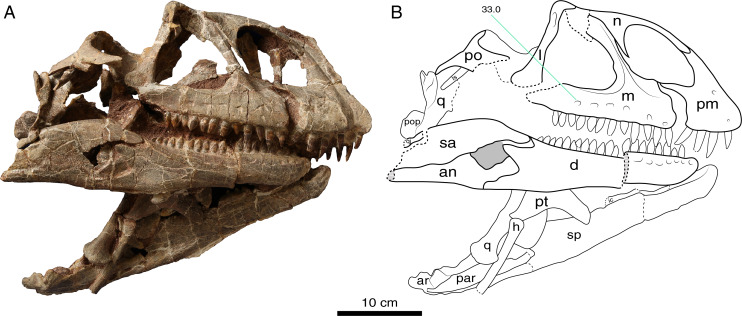
Photograph (A) and interpretative line drawing (B) of the cranium of *Lishulong wangi* gen. et sp. nov. in right lateral view. Abbreviations: an, angular; ar, articular; d, dentary; h, hyoid; ic, intercoronoid; j, jugal; l, lacrimal; ls, laterosphenoid; m, maxilla; n, nasal; par, prearticular; pm, premaxilla; po, postorbital; pop, paraoccipital process; pt, pterygoid; q, quadrate; qj, quadratojugal; sa, surangular; sp, splenial. Dark grey fills represent the external mandibular fenestra and dashed lines represent fracture. (Photo credit: Wei Gao).

**Figure 3:**
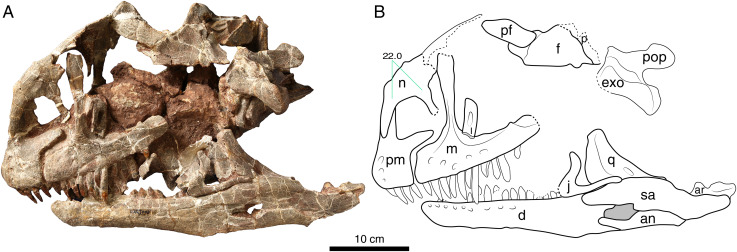
Photograph (A) and interpretative line drawing (B) of the cranium of *Lishulong wangi* gen. et sp. nov. in left lateral view. Abbreviations: ar, articular; d, dentary; exo, exoccipital-opisthotic complex; f, frontal; j, jugal; l, lacrimal; m, maxilla; n, nasal; p, parietal; pf, prefrontal; pm, premaxilla; pop, paraoccipital process; q, quadrate; sa, surangular. Dark grey fills represent the external mandibular fenestra and dashed lines represent fracture. (Photo credit: Wei Gao).

**Figure 4:**
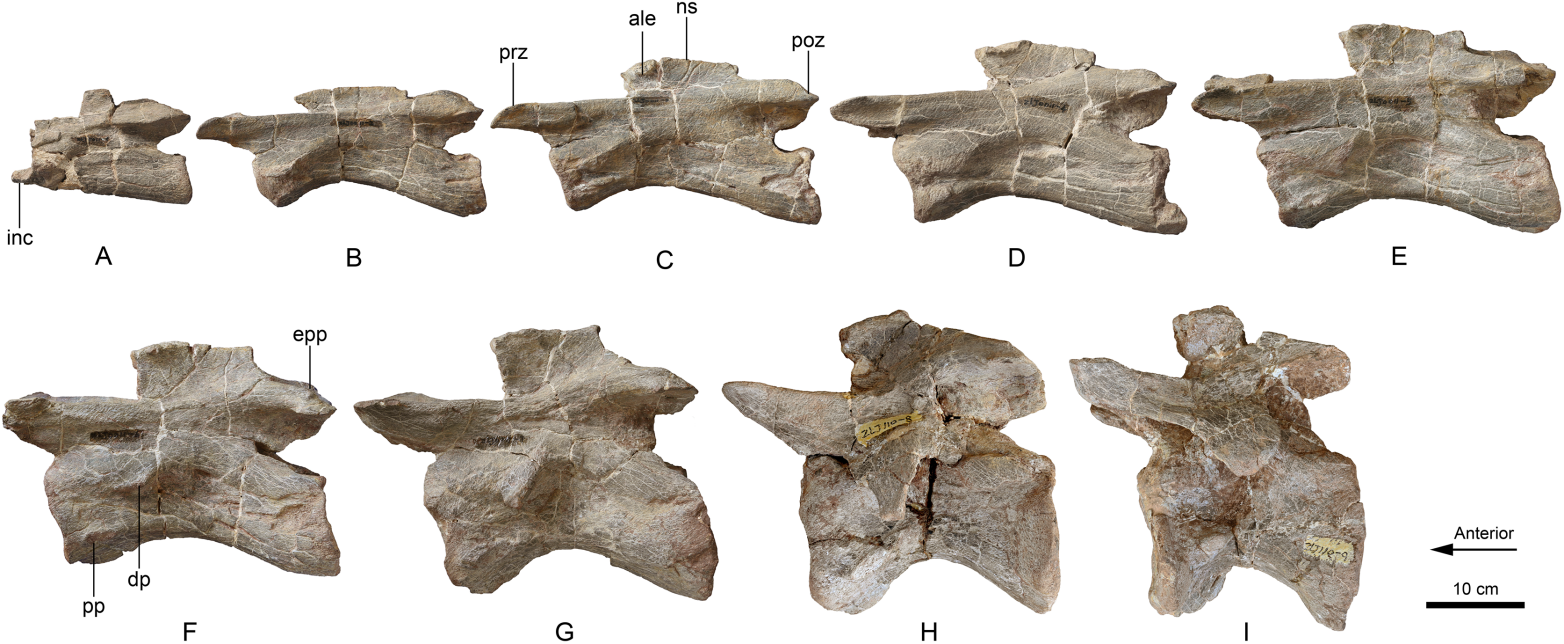
Axis and cervical vertebrae 3–10 (A–I) of *Lishulong wangi* gen. et sp. nov. in left lateral view. Abbreviations: ale, anterolateral expansion; dp, diapophysis; epp, epipophyses; inc, intercentrum; poz, postzygapophysis; pp, parapophysis; prz, prezygapophysis. (Photo credit: Wei Gao).

**Figure 5:**
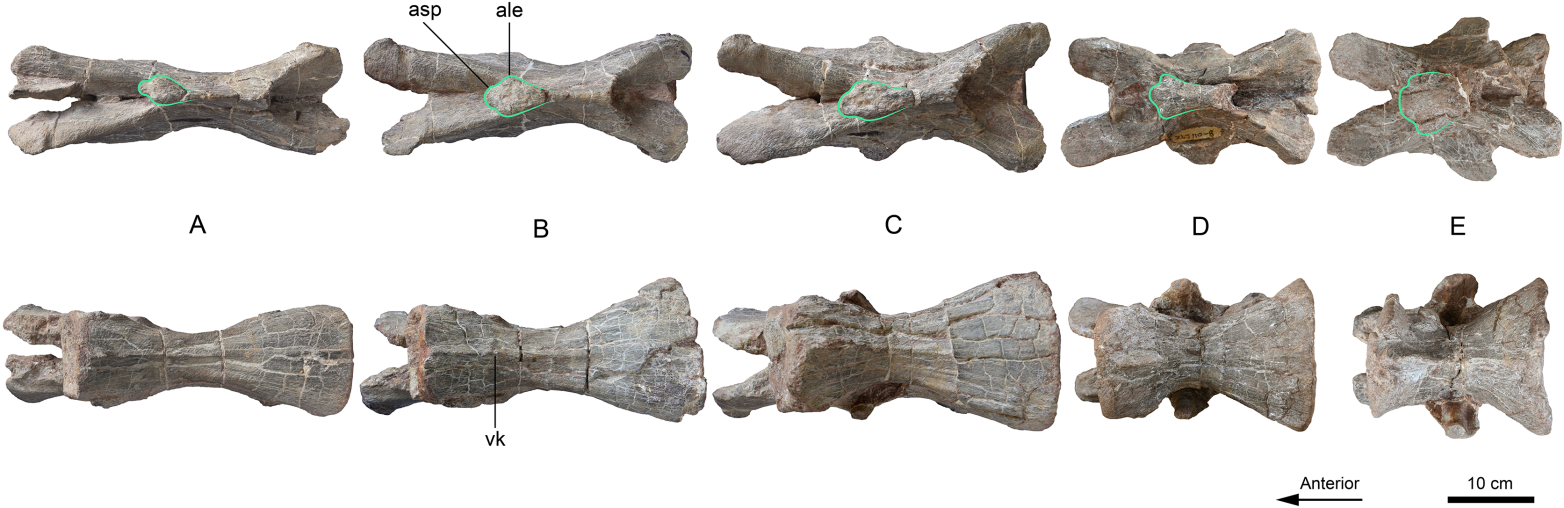
Cervical vertebrae 6–10 (A–E) of *Lishulong wangi* gen. et sp. nov. in dorsal and ventral views. Abbreviations: ale, anterolateral expansion; asp, anterior spur-like projection; vk, ventral keel. (Photo credit: Wei Gao).

**Holotype:** LFGT-ZLJ0011. An associated partial skeleton that includes the cranium and mandible, and nine cervical vertebrae (axis and C3–C10) (Figs. 2–5).

**Type locality and horizon:** The specimen was discovered near the Jiudu Village in Konglongshan Town (formerly named Chuanjie Township), Lufeng County, Yunnan Province, China; and the upper-middle part of the Shawan Member of the Lufeng Formation (Fang et al., 2000), Lower Jurassic. Magnetostratigraphic analyses (Cheng et al., 2004; Huang et al., 2005) revealed the age to be Early Jurassic (late Sinemurian–Toarcian).

**Etymology:** The generic name is from ‘Lishu’ (chestnut tree in Chinese spelling), the name of the locality where the specimen was found, and ‘long’ refers to a dragon (in Chinese spelling); this specific epithet is in honor of Mr. Zheng-Ju Wang, for his great contributions to the early discoveries of vertebrate fossils from Lufeng.

**Differential diagnosis:** A large non-sauropodan sauropodiform dinosaur with the following unique combination of character states (autapomorphies are indicated by an asterisk): width of the anteroventral process of nasal at its base less than that of its anterodorsal process*; size of the neurovascular foramen at the posterior end of the lateral maxillary row not larger than the others*; shape of the supraoccipital is semilunate and wider than high in posterior view; height to length ratio of the dentary greater than 0.2; lingual concavities of the teeth present; lateral expansion at the anterior region of the dorsal surface of the cervical neural spines*.

### Description

#### Skull

The skull of LFGT-ZLJ0011 is relatively large with thin vertically supportive bones. It seems to have undergone severe transverse compression, resulting in the distortion of its dorsal and palatal elements (Figs. 2–4). However, the two lateral sides of the skull are essentially intact, except for some missing bones in the orbital and temporal regions, such as the postorbital, jugal, quadratojugal and squamosal, which were displaced from their life position or even lost (Figs. 2 and 3). The skull is low and elongated in lateral view with an anteroposterior length of 40 cm (based on the left mandibular length), making it the largest cranial material recovered from the Lufeng Formation, while the previously largest is the skull of *Jingshanosaurus*, which is about 35 cm long (Zhang et al., 2020).

Most of the skull openings of LFGT-ZLJ0011 are broken; therefore, the shapes of the orbit, supratemporal, and infratemporal fenestra are difficult to deduce with certainty. The right external naris is large with the anteroposterior length accounting for approximately 22% of the total skull length, which is similar to the 20% ratio of *Jingshanosaurus* (Zhang et al., 2020), but that of *Yunnanosaurus* is only 10% (Barrett et al., 2007). This opening is subtriangular in shape and bordered by the premaxilla (anteroventrally), maxilla (posteriorly), and nasal (dorsally) (Fig. 2). The posterior margin of this opening is located posterior to the premaxilla-maxilla suture; however, it is anterior to the anterior margin of the antorbital fenestra, which is different from that of *Jingshanosaurus* (Zhang et al., 2020). The posteroventral corner of the external naris is at an obtuse angle, resembling that of *Yizhousaurus* (LFGT-ZLJ0033); however, this angle is acute in *Yunnanosaurus* (IVPP V 20/NJGM 004546) and forms a right angle in *Jingshanosaurus* (LFGT-ZLJ0113 and CXM-LT9401). In right lateral view, the antorbital fenestra appears to form an approximate equilateral triangle (Fig. 2), which is similar to that of *Lufengosaurus* (IVPP V 15); however, it is broader anteroposteriorly than those of *Yizhousaurus* (Zhang et al., 2018). This opening of LFGT-ZLJ0011 is set within a triangular antorbital fossa, which is deeply impressed on the maxilla anteroventrally (Figs. 2 and 3).

**Premaxilla**. The premaxilla has a subtriangular shape in lateral view, forming the anterior end of the snout as well as the anteroventral margin of the external naris (Figs. 2 and 3). The premaxilla contacts the maxilla posteriorly and the nasal dorsally. It projects two processes, the anterodorsal nasal process and the posterolateral maxillary process, arising from the main body. The lateral surface of the premaxillary body is slightly convex, while its ventral margin is level with that of the maxilla. On the anterolateral surface of the premaxillary body, three neurovascular foramina are present in a subvertical row, which are more visible on the left premaxilla (Fig. 3) and at least two can be identified on the right side (Fig. 2). This foramina arrangement is similar to those of *Adeopapposaurus* (Martínez, 2009), *Jingshanosaurus* (CXM-LT9401) and *Massospondylus* (Sues et al., 2004).

The nasal process of the premaxilla extends posterodorsally and forms most of the anterior margin of the external naris. In lateral view, this process is anteroposteriorly expanded at its base where it joins the premaxillary body and posterodorsally tapers and elongates to its distal end (Fig. 2). The inflection at the base of the premaxillary nasal process is absent in LFGT-ZLJ0011, different from those of *Jingshanosaurus* (CXM-LT9401) and *Melanorosaurus* (NM QR3314).

The maxillary process extends posteriorly from the dorsal margin of the premaxilla, forming the ventral margin of the external naris. This process is a flat strap structure that overlaps the anterodorsal region of the premaxillary process of the maxilla (Figs. 2 and 3). In lateral view, the dorsoventral height of the maxillary process accounts for approximately 30% of the total premaxillary body height. The ventral margin of the maxillary process and the posterior margin of the premaxillary body form an ‘L’-shaped suture between the premaxilla and maxilla, and define a conspicuous subnarial foramen exposed externally below the maxillary process (Fig. 3).

**Maxilla.** The posterior-most regions of both maxillae are fractured, lacking posterior contacts with the jugal and lacrimal. The remaining maxilla contacts the premaxilla anteriorly, and the lacrimal and nasal dorsally, forming the posteroventral margin of the naris, as well as the anterior and ventral margins of the antorbital fenestra. The maxilla has a triradiate lateral profile comprising three processes: the anterior premaxillary, the posterior jugal, and the posterodorsal ascending processes (Figs. 2 and 3). In lateral view, the maxilla is long and straight anteroposteriorly, with its dorsal and ventral margins parallel to each other. The maxilla is at its dorsoventral highest anteriorly and tapers towards its posterior end. A linear row with at least six neurovascular foramina is present on the lateral surface of the right maxilla, most of which are ventro- or postero-laterally oriented (Fig. 2). They are evenly spaced and similarly sized, and the posterior-most one is not larger than the anterior ones (Fig. 2), which is characterized and different from other non-sauropodan sauropodomorphs (Plateosauridae and Massospondylidae) with a distinctly larger foramen at the posterior end. There is no evidence of a lateral maxillary ridge, which differs from those of *Lufengosaurus* (Barrett & Upchurch, 2005) and *Melanorosaurus* (Yates, 2007). The alveolar region of the maxilla is not developed, indicating that the ‘lateral plate’ is absent; however, it is preserved in *Aardonyx* (Yates et al., 2010) and *Yizhousaurus* (Zhang et al., 2018).

The premaxillary process is sub-rectangular and the shortest of the three processes. The anterodorsal region of this process is overlapped by the maxillary process of the premaxilla (Fig. 3). Its anteroposterior length is a little greater than its dorsoventral depth, which is different from *Jingshanosaurus* (both LFGT-ZLJ0113 and CXM LT-9401) that has a shorter premaxillary process of the maxilla. The dorsal surface of the premaxillary process is slightly inclined and orientated dorsolaterally, forming the maxillary region of the narial fossa.

The ascending process of the maxilla is slender and much less anteroposteriorly expanded than in most Lufeng sauropodomorphs, such as *Lufengosaurus* (IVPP V 15), *Yunnanosaurus* (IVPP V 20/NJGM 004546), *Xingxiulong* (LFGT-D0003) and *Yizhousaurus* (LFGT-ZLJ0033). Although the maxillary ascending processes of *Jingshanosaurus* (LFGT-ZLJ0113) are not well preserved, those of CXM LT-9401 are also anteroposteriorly expanded, especially at the top. This process arises from a point approximately one-third of the anteroposterior length of the maxilla and gradually inclines posterodorsally (Figs. 2 and 3). It forms the posterior margin of the external naris, thereby separating the maxillary processes of the premaxilla and nasal. The ascending process possesses a distinct ridge that runs along the midline of its entire lateral surface (Fig. 2). The anterodorsal region of the ascending process is overlapped by the ventrolateral process of the nasal, and the posterodorsal region contacts the lacrimal. The posteroventral surface of the ascending process is excavated, forming a thin subtriangular medial lamina of the antorbital fossa, as in *Adeopapposaurus* (Martínez, 2009), *Jingshanosaurus* (CXM-LT9401), *Massospondylus* (Sues et al., 2004), and *Mussaurus* (Pol & Powell, 2007), different from the anteroposteriorly broad medial lamina of *Coloradisaurus* (Apaldetti et al., 2014), *Lufengosaurus* (IVPP V 15), *Melanorosaurus* (Yates, 2007), *Plateosaurus* (Prieto-Márquez & Norell, 2011), *Riojasaurus* (Bonaparte & Pumares, 1995) and *Unaysaurus* (Leal et al., 2004).

**Nasal**. Although both nasals are preserved, the right one is better preserved than the left; however, the midline suture between the two is difficult to determine owing to the compression. The nasal comprises an anterior premaxillary process, a ventral maxillary process, and a posterior process (Fig. 2). In lateral view, the dorsal and lateral surfaces of the nasal are slightly convex without a depression posterior to the naris, which is present in *Adeopapposaurus* (Martínez, 2009), *Lufengosaurus* (IVPP V 15), *Massospondylus* (Sues et al., 2004) and *Plateosaurus* (Prieto-Márquez & Norell, 2011).

The anteriorly extending premaxillary process forms the anterodorsal margin of the external naris. The mediolateral width of the premaxilla process of the nasal at its base is less than the anteroposterior width of the maxilla process at its base (Fig. 3), which is considered to be an autapomorphy of *Lishulong*, given that this condition is opposite in almost all non-sauropodan sauropodomorphs. The premaxillary process gradually curves and tapers anteroventrally to a lap joint that contacts the premaxillary nasal process medially (Figs. 2 and 3). The posterior process is anteroposteriorly shorter than the premaxillary process, and its posterior region is broken. It extends posteriorly to contact the anterior end of the frontal and the medial surface of the prefrontal; however, these three elements are disarticulated (Fig. 3).

In lateral view, the maxillary process of the nasal is a subtriangular sheet of bone that tapers sharply as it extends ventrally (Fig. 2). It contacts the anterolateral surface of the ascending process of the maxilla and extends for approximately half of the dorsoventral height of the ascending process, as present in many early sauropodomorphs (*e.g*., *Coloradisaurus*, *Lufengosaurus*, *Massospondylus* and *Yunnanosaurus*). The posterodorsal region of the maxillary process partially contributes to the anterodorsal margin of the antorbital fossa and overlaps the lacrimal with short contact.

**Lacrimal.** The right lacrimal is well-preserved and undistorted; however, only a third of the dorsal region of the left one is preserved, which falls on the anteroventral corner of the antorbital fenestra (Fig. 3). The lacrimal initially contacts the maxilla and nasal anterodorsally, the prefrontal posterodorsally, and the maxilla and jugal ventrally. It contains an anteroventrally directed short process and a posteroventrally oriented main shaft. In lateral view, the two rami meet at approximately 90° to each other, forming an inverted ‘L’-shape (Fig. 2). The anterior process is anteroposteriorly short with a rounded vertex, forming a small, triangular shelf that overhangs the posterodorsal corner of the antorbital fenestra. The main shaft of the lacrimal is an anteroposteriorly thin and dorsoventrally tall structure with a deep groove posteriorly opening lateral to it, indicating the posterior opening of the foramen for the nasolacrimal duct (Figs. 2 and 3). Although the morphology of the lacrimal is similar to that of other Lufeng taxa, that of *Lishulong* is significantly more elongated and thinner with a smaller dorsal process. The ventral region of the lacrimal shaft is posteroventrally expanded, forming a subtriangular, rugose surface for contacting the anterior process of the jugal.

**Prefrontal.** Only part of the left prefrontal is preserved and displaced posteriorly from its natural position. Logically, the prefrontal contacts the nasal anteriorly, the lacrimal anteroventrally, and the frontal posteromedially. The remaining region of the prefrontal is dorsoventrally flattened with a tab-like structure exposed on the skull roof (Fig. 3). The posterior region of the prefrontal has an overlapped contact with the frontal, which borders it posteriorly and medially.

**Frontal**. The right frontal is also missing, and the left one is incompletely preserved. The frontal is a sub-trapezoidal, thick plate that is anteroposteriorly longer than it is transversely wide (Fig. 3), although its posterior end is broken, different from the wider frontal of *Yizhousaurus* (LFGT-ZLJ0033). The anterior region of the frontal is narrow and tab-like with a rounded distal margin, overlapped by the prefrontal, and its suture with the nasal cannot be determined accurately due to poor preservation. The dorsal surface of the frontal is gently concave, bearing a shallow depression. The lateral margin of the frontal is thickened, while the posterior margin of the frontal is linear and contacts the anterior margin of the parietal.

**Postorbital**. The right postorbital is preserved with the ventral process fractured. It is a triradiate bone that comprises the ventral jugal process, as well as the anteriorly and posteriorly directed dorsal processes (Fig. 2). Every process separates from an angle of approximately 120° to the other two. The anterodorsal process of the postorbital is dorsoventrally compressed and mediolaterally widened into a tab-like structure. The dorsal surface of the anterodorsal process is convex. The posterodorsal process of the postorbital is typically shorter than the other two processes, it has a subtriangular tip and tapers posteriorly, where it contacts the squamosal.

**Jugal**. The posterior-half region of the left jugal is preserved; however, it is disarticulated from the buccal area. It has two posterior processes left: the posterodorsal postorbital process and the posterior quadratojugal process, together forming a sideways ‘V’-shaped opening directed posteriorly (Fig. 3), which sets an angle of approximately 60 degrees, resembling that of *Yunnanosaurus* (Barrett et al., 2007), but smaller than those in *Plateosaurus* (80 degrees or 95 degrees; Prieto-Márquez & Norell, 2011) and *Xingxiulong* (80 degrees), larger angle than those in *Lufengosaurus* (50 degrees; Barrett, Upchurch & Wang, 2005), *Massospondylus* (40 degrees; Chapelle & Choiniere, 2018), *Mussaurus* (50 degrees; Pol & Powell, 2007). The quadratojugal process is slender and elongated, and tapers out posteriorly as it contacts the quadratojugal. The postorbital process projects posterodorsally from the main body of the jugal. It is relatively short and slightly curved, dorsally tapering to contact the ventral process of the postorbital.

**Quadrate**. Both quadrates are preserved, and the left one is more complete than the right one, even though the former is dislocated over a distance (Fig. S1A). The quadrate forms the posterolateral margin of the skull that would articulate with the mandible. The quadrate comprises a quadrate head, a main shaft, a lateral quadratojugal process, and a medial pterygoid process. In lateral view, the quadrate head is subtriangular and mediolaterally compressed (Fig. 3). The main shaft is robust and slightly bowed anteriorly with its posterior surface shallowly excavated along its length. The posterior margin of the quadrate shaft is thickened into a ridge-like structure that extends from the quadrate head to the quadrate condyles. The quadrate foramen is present below the dorsal half of the posterior surface, lateral to the ridge of the main shaft (Figs. 3 and 4A). Distally, the shaft expands both anteroposteriorly and transversely to form the articulation that contacts the mandible. This articular region is divided into two semicircular lateral and medial condyles by a deep intercondylar groove. The posteromedial margin of the medial condyle is more ventrally located than that of the lateral one.

The pterygoid process of the quadrate extends anteriorly as a mediolateral sheet element that occupies more than two-thirds of the dorsal quadrate shaft. The dorsal region of the pterygoid process grades gradually into the quadrate head (Fig. 3). It forms an extensive articulation with the quadrate process of the pterygoid along its entire anterior margin. The quadratojugal process extends anterolaterally from the dorsal half of the quadrate shaft, at an angle of approximately 90° with the pterygoid process. The two processes of the quadrate are set at an angle of less than 90° and separated by a large concave area on the anterior surface of the quadrate (Fig. 2).

**Pterygoid.** The incomplete left pterygoid is exposed in the right lateral and ventral views (Figs. 2 and 4B). The pterygoid makes up most of the posterior region of the palate and contacts the quadrate posterolaterally. The pterygoid comprises three sections: the central region, the anterior palatine process, and the quadrate process. The palatine process of the pterygoid is fractured together with the anterior palatal bones, including the vomer and palatine. The central region of the pterygoid is complex, it projects a short medial process to contain the basipterygoid process, and a lateral flange anterior to the quadrate process (Fig. S1B). The lateral flange is robust and contacts the ectopterygoid dorsally. The quadrate process is a large lamina that curves posterolaterally from the central region of the pterygoid. It is concave along its medial surface and convex laterally, and overlapped by the pterygoid process of the quadrate medially.

**Supraoccipital**. The supraoccipital is well-preserved and sloped anterodorsally. In posterior view, it has a roughly pentagonal outline and it is transversely wider than dorsoventrally high (Fig. S1), which differs from the typical higher supraoccipitals present in most non-sauropodan sauropodomorphs, except *Ngwevu* (Chapelle et al., 2019). The supraoccipital contacts the exoccipital ventrolaterally, its lateral margin contributes to the base of the paroccipital processes of the exoccipitals. The posterodorsal surface of the supraoccipital contains a low and rounded median ridge that extends dorsoventrally along the midline of the supraoccipital. On the dorsal end of the median ridge is a notch, which is presumed to be the post-parietal fenestra (Fig. S1A). The ventral margin of the supraoccipital forms two prominent protuberances, forming the dorsolateral margin of the foramen magnum.

**Exoccipital-opisthotic.** The exoccipital is relatively complete and fused to the opisthotic as a complex element. It forms the lateral margin of the foramen magnum and the dorsolateral region of the occipital condyle (Fig. S1A). Each exoccipital contacts the ventral region of the supraoccipital and the dorsal region of the basioccipital. The paroccipital process with a blunt, rounded distal end projects posteriorly and ventrolaterally (Fig. 3). This process has a straight or slightly convex dorsal margin and a concave ventral margin.

**Basioccipital.** The basioccipital is completely preserved and undistorted. It contacts the exoccipital dorsally and the basisphenoid anteriorly to form the ventral margin of the foramen magnum and the posterior region of the braincase (Fig. S1). The basioccipital expands posteriorly to form the occipital condyle, which appears as a sub-crescent in posterior view. In ventral view, it appears separated from the basal tubera by a constricted neck. On the ventral side of the condylar neck, there is a shallow vertical fissure. The basioccipital contacts part of the basisphenoid along its anterior surface; however, the suture is sinuous, and only an anteroposteriorly compressed ridge signifies the remnant of the basal tuberae.

#### Mandible

Both mandibles of LFGT-ZLJ0011 are present and complete in general, only the posterior region of the right mandibular ramus is broken and not as well preserved as the left one. The mandible is slender and elongated, with a long retroarticular process (Fig. 3). The external mandibular fenestra has a sub-elliptical outline in lateral view, and its anteroposterior length is approximately 11% of the total mandibular length, similar to the condition of *Jingshanosaurus* (LFGT-ZLJ0113), contrary to the relatively large size in most non-sauropodan sauropodomorphs. It is bounded anteriorly by the dentary, posterodorsally by the surangular, and posteroventrally by the angular. The coronoid eminence is developed, similar to those of *Lufengosaurus* (IVPP V 15) and *Yunnanosaurus* (IVPP V 20/NJGM 004546), which is approximately twice the height of the tooth-bearing part of the dentary. The jaw articulation is on the same level as the dentary tooth row, resembling the condition in *Yunnanosaurus* (IVPP V 20/NJGM 004546) and *Xingxiulong* (LFGT-D0003).

**Dentary**. The dentary makes up approximately more than half the total mandibular length. It maintains an almost constant height along its length with a slight increase in depth towards its posterior region (Figs. 2 and 3). The ratio of the height to length of the dentary is about 0.23, which is higher than that of other early sauropodomorphs (<0.2); however, it is lower than the ratio of 0.28 of *Jingshanosaurus* (LFGT-ZLJ0113). In lateral view, the anterior end of the dentary is subtriangular with a rounded tip. The lateral surface of the dentary is gently convex and the dorsal margin is pierced by a linear row of subequal neurovascular foramina (Figs. 2 and 3). Posteriorly, the dentary delimits the anterior margin of the external mandibular fenestra, eventually splitting into two processes that contact the surangular posterodorsally and the angular posteroventrally.

**Surangular**. In lateral view, the surangular is an elongated, sigmoidal element (Fig. 3). The lateral surface of the surangular is slightly convex, with its ventral margin forming most of the posterodorsal margin of the external mandibular fenestra. The surangular reaches its dorsoventrally largest depth at the level of the coronoid eminence, its dorsal margin thickened and oriented transversely to form the dorsal region of the coronoid. The medial surface is concave, forming a groove on the medial side of the mandible. The surangular contacts the dentary and splenial anteriorly, the angular ventrally, and the articular posteromedially to form the retroarticular process. The retroarticular process is finger-like with a concave dorsal surface at the jaw joint region.

**Angular**. The angular is a strap-like element that makes up the posteroventral region of the mandible. It has a broad contact with the surangular dorsally while articulating with the dentary anteriorly, and its dorsal margin forms the ventral border of the external mandibular fenestra (Figs. 2 and 3). In ventral view, the angular meets the prearticular along an almost straight anteroposterior suture with its medial surface being overlapped by the latter (Fig. S1).

**Intercoronoid**. There is some indication of an anteroposteriorly elongated intercoronoid that is located on the dorsomedial margin of the left mandible (Fig. 2). The intercoronoid is a thin bony plate that covers the lingual alveolar of the dentary. It is approximately 6 mm with a constant dorsoventral depth.

**Splenial.** The splenial is a flat, laminar bone that covers the dentary medially with its dorsal margin partially overlapping the intercoronoid (Fig. 2). The anterior region of the splenial is damaged, and no splenial foramen is visible, whereas the rest of it splits into two processes posteriorly. The sutures with the dentary, angular and prearticular could not be determined.

**Prearticular**. The prearticular is preserved and exposed in medial view (Fig. 2). It is an elongated, strap-like element that forms the medial region of the mandible’s posteroventral area. The dorsal surface of the prearticular is a shallowly concave midsection. Its posterior region comprises a portion of the retroarticular process medial surface and covers the medial surface of the articular.

**Articular**. The articular is a small block-like bone positioned dorsally to the prearticular and contacts the surangular laterally (Fig. 2). The dorsal surface of its anterior region is concave both anteroposteriorly and transversely for articulation with the quadrate. The articular has a blunt posterior tip that forms the medial region of the retroarticular process. There is a small, tab-like process directed medially posterior to the mandibular cotyle, this is also preserved in *Xingxiulong* (LFGT-D0003) and *Jingshanosaurus* (LFGT-ZLJ0113).

**Ceratobranchial.** A part of the left ceratohyal is preserved under the quadrate, medial to the left mandibular ramus (Figs. 2 and 4). It is elongated and rod-like with its anterior end expanded into a protuberance, and its posterior end is almost constant in diameter and slightly curves medially.

#### Dentition

In general, most of the teeth of LFGT-ZLJ0011 are well preserved, except for some left dentary teeth that are apically broken (Fig. 3). The morphology of the premaxillary teeth is nearly identical to that of the maxillary teeth. All the upper jaw teeth are more slender with apicobasal elongated crowns and appear to be more circular in the cross-section, leading to the inapparent mesiodistal constriction of the roots at the base. These teeth are linearly placed without overlapping crowns between adjacent teeth, with gaps visible between them in lateral view (Fig. 2; Fig. S2), instead of the typical imbricated arrangement. The crowns are apicobasally longer than they are mesiodistally wide. The tooth crowns display no distal recurvature, differing from the distally recurved crowns of *Jingshanosaurus* (LFGT-ZLJ0113). Coarse serrations are restricted to the apical half of the crown on both the mesial and distal carinae. The number of premaxillary teeth is clearly determined to be four, they are apicobasally higher than all the rear teeth. To determine the exact number of maxillary teeth is challenging because the posterior tooth rows are poorly preserved; however, at least 12 teeth are preserved on either side of the maxilla (Figs. 2 and 3). The labial surface of the tooth crown is slightly convex, whereas the lingual surface is slightly concave, which is different from the relatively flat lingual surfaces in other non-sauropodan sauropodomorphs and the highly concave condition in sauropods. The enamels of the teeth are smooth with gracile longitudinal striations, and finely wrinkled enamel has also been described in *Jingshanosaurus* (LFGT-ZLJ0113) and *Irisosaurus* (de Fabrègues et al., 2020). There is no indication of any genuine wear facets. The lower tooth row contains at least 20 teeth on the left side (Fig. 3), and the dentary teeth are smaller in size than the upper teeth.

#### Cervical vertebrae

Nine cervical vertebrae are preserved in the holotype (LFGT-ZLJ0011), including the axis along with eight associated cervicals (C3–C10); therefore, with the missing atlas, *Lishulong* should possess at least ten cervical vertebrae originally, which is typical condition for non-eusauropod sauropodomorphs. The neck is proportionally enlarged and elongated along with the skull. All the vertebrae are highly complete, and the centra are typically amphicoelous and solid inside without the evidence of camerae.

The well-preserved axis is much shorter than the postaxial cervical vertebrae. The centrum of the axis is slightly compressed laterally, and 3.4 times longer than it is high, which is slightly greater than that of *Lufengosaurus* (Young, 1941a), *Jingshanosaurus* (Zhang & Yang, 1995), and *Xingxiulong* (Wang, You & Wang, 2017). Its dorsal surface is almost flat along the neural canal, and its ventral surface bears a markedly longitudinal keel that runs along the anterior two-thirds of its ventral length. An incompletely preserved intercentrum is fused to the ventral region of the anterior surface of the axial centrum (Fig. 4); however, it is not broader than the width of the centrum in anterior view, which is different from that of *Yizhousaurus* (Zhang et al., 2018). The intercentrum is somewhat broken, its anteroposterior length is approximately one-eighth of the total central length. The neural canal is large, occupying more than half the anterior surface of the centrum. The posterior surface of the centrum is circular and concave. The neural arch is tightly fused to the centrum extending along almost its entire length (Fig. 4). The neural spine is damaged in its anterior region, and the remaining posterior half is subtriangular in dorsal view. The diapophyses are absent, whereas the parapophyses are situated ventrolaterally in the anterior region of the centrum, presenting as anteroposteriorly elongated tubercles (Fig. 4). The prezygapophyses are also inconspicuous on the anterolateral area of the neural arch. The postzygapophyses extend flush with the posterior surface of the centrum (Fig. 4). The epipophyses are developed as moderate ridges on the dorsal surface of the postzygapophyses, extending along their entire length.

**Figure 6:**
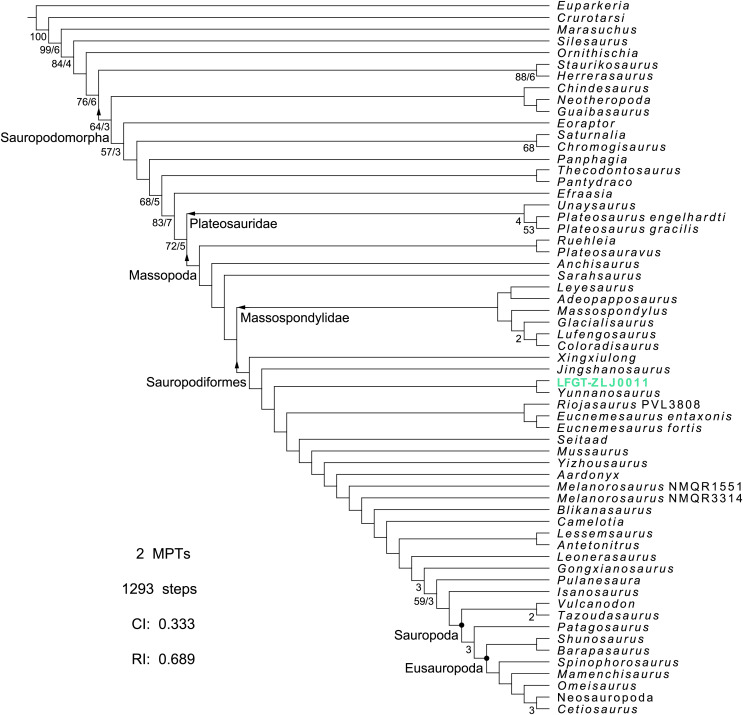
Complete strict consensus tree of phylogenetic analysis depicting the position of *Lishulong wangi* gen. et sp. nov. Numbers below the nodes represent bootstrap values higher than 50% (left) and bremer support values higher than 1 (right). Abbreviations: CI, consistency index; MPTs, most parsimonious trees; RI, retention index.

The eight postaxial cervical vertebrae of LFGT-ZLJ0011 are completely preserved. All the cervical centra are more elongated and robust than those of other Lufeng sauropodomorphs, even more than twice the length of each cervical of *Lufengosaurus* (Young, 1941a), *Yunnanosaurus* (Young, 1942) and *Xingxiulong* (Wang, You & Wang, 2017). The third cervical has a remarkably elongated centrum, which is approximately 1.4 times the length of the axial element with a centrum length/height ratio is approximately 4.6. The abrupt elongation of cervical 3 is also observed in *Coloradisaurus* (Apaldetti, Pol & Yates, 2013), *Lufengosaurus* (Young, 1941a), and *Yizhousaurus* (Zhang et al., 2018); however, it differs from *Yunnanosaurus* (Young, 1942), in which the third cervical is only one-third longer than the axis. The centra of cervicals 4 to 7 gradually increase their length to the maximum, whereas cervicals 8 to 10 are progressively shorter. All these centra are compressed both laterally and ventrally with remarkably developed ventral keels, which become more noticeable in the posterior cervical vertebrae (Figs. 4 and 6). The neural arches are strongly fused to the centra with their heights lower than those of their respective centra (Fig. 4). The articular surfaces of the centra are sub-circular with shallowly concave anterior surfaces and deeply concave posterior surfaces, which is typically present in other early sauropodomorphs. The posterior surfaces of the centra in the anterior cervical vertebrae are subequal in height and width; however, the centra of cervicals 6 to 10 are not higher than they are wide, indicating dorsoventral compression of the posterior centra. The neural spines are low and elongated, becoming higher and thicker from the anterior to posterior cervical vertebrae. The anteroposterior length of the neural spines progressively increases up until cervical 7, and then becomes shorter posteriorly. The anterior ends of the dorsal surface of the neural spines are laterally expanded as two protuberances, and the posterior region appears as transversely thin plates in dorsal view, forming an anteroposterior sub-rhombic at the anterior half of the spines (Fig. 5), instead of laterally expanded tables at the midlength of the dorsal surface of the neural spines. This anterolateral expansion of the cervical neural spines is unique in LFGT-ZLJ0011; therefore, it is treated as one of the autapomorphies of *Lishulong*. The diapophyses are weakly developed in the anterior and middle cervicals, presenting as elongated ridges close to the anterior margins of the centra, in posterior vertebrae they form aliform flanks that become progressively larger (Fig. 4). The parapophyses are present as sub-elliptical protuberances situated at the anteroventral region of the lateral surfaces of the centra, except in the last two vertebrae in which they shift progressively to a more posterior position. The zygapophyses of most cervical vertebrae extend horizontally with flat articular facets; however, they are slightly upturned approximately 20° with respect to the centra in cervicals 9 and 10 (Fig. 4). The prezygapophyses develop distinct ventrolateral ridges, so the centroprezygapophyseal laminae are robust in anterior view, while the prezygodiapophyseal laminae are present only in both cervicals 9 and 10, not as developed as in derived sauropodiforms. Along with the cervical series, the prezygapophyses extend far beyond the anterior surface of the centra; however, the postzygapophyses are short and do not overhang to the posterior margin of the centra. The epipophyses are low ridges that extend along the dorsal surface of the postzygapophyses (Fig. 4) with their posterior ends decreasing in height and not overhanging the posterior edge of the postzygapophyses.

## Discussion

### Phylogenetic analysis

In order to assess the phylogenetic relationships of *Lishulong wangi* among contemporary sauropodomorph taxa, we scored it using the phylogenetic dataset reported by Zhang et al. (2018). We also accept the 27 cranial character scorings for *Jingshanosaurus* revised by Zhang et al. (2020). This analysis resulted in two MPTs with the shortest length of 1,292 steps (CI: 0.333; RI: 0.689). The strict consensus tree produced from this phylogenetic analysis exhibits a relatively good resolution with no polytomies (Fig. 6). *Lishulong* is recovered as an early-diverging sauropodiform member, sister taxon of *Yunnanosaurus*, occupying the position closest to the Sauropodiformes node with *Xingxiulong* and *Jingshanosaurus*. The node of *Lishulong* + *Yunnanosaurus* is supported by three unambiguous synapomorphies: web of bone spanning junction between anterior and ventral rami of the lacrimal obscuring the posterodorsal corner of the antorbital fossa (char. 41, state 1); position of the jaw joint not lower than the level of the dorsal margin of the dentary (char. 94, state 0); arrangement of the teeth within the jaws linearly placed and crowns not overlapping (char. 109, state 0).

The phylogenetic analysis yielded well-supported Plateosauridae and Massospondylidae across all trees with the Bremer values over 2, and other clades near the Sauropoda node (Fig. 6). However, the cladistic positions of many non-sauropodan sauropodiforms are labile in different matrices, which is likely due to missing scores of some of the more poorly preserved specimens (*e.g*., *Blikanasaurus* and *Camelotia*), ambiguous character definitions, or uncertainty of source specimen referred to for scoring in the previous matrix (*e.g*., *Riojasaurus* and *Melanorosaurus* treated as separated individuals). All the Chinese specimens involved in the current analysis belong to Sauropodiformes, except *Lufengosaurus*, while *Yizhousaurus* is recovered closer to ‘sauropod-like’ forms. *Lishulong* and another three Lufeng genera occupy the transitional position between the early- and late-branching of non-sauropodan sauropodomorphs. Therefore, further descriptions and revisions are important to better represent phylogenetic relationship statements.

### Morphological comparison

Based on the current fossil records, *Lishulong wangi* is the largest sauropodomorph from the Early Jurassic epoch in China, and is considered morphologically mature according to the vertebral morphology and completely fused centra and neural arches (Griffin et al., 2021). *Lishulong* is distinguished from other non-sauropodan sauropodomorphs with transversely wider supraoccipital and lingual concave teeth that are present in derived sauropods.

*Lufengosaurus huenei* is the only named member of Massospondylidae among those Lufeng specimens, which was originally described by Young (1941a). Other massospondylid material has been described by Zhao et al. (2024), which is very similar to *Lufengosaurus* in morphology. The cranial anatomy of *Lufengosaurus* has been re-examined with amended diagnoses by Barrett, Upchurch & Wang (2005), wherein four autapomorphies were proposed for *Lufengosaurus*: distinct tuberosity on lateral surface of ascending process of maxilla; low boss on central portion of jugal at junction of the three jugal processes; prominent boss on dorsal surface of anterolateral process of parietal; and presence of a ridge on the caudal part of the lateral surface of maxilla. The first three features focus on the various bosses. The maxillary and jugal bosses are more distinct on the right side than the left, probably due to preservation, because the skull of *Lufengosaurus* suffered dorsoventral compression in personal observation. The parietal boss actually indicates the junction of the parietal, frontal, and postorbital, which is more prominent than in other sauropodomorphs. In addition to these bosses, *Lishulong* can still be distinguished from *Lufengosaurus* by the absence of the ridge on its posterolateral surface, anteroposteriorly narrower ascending process of its maxilla, and its transversely wider supraoccipital.

*Xingxiulong chengi* only has one autapomorphic characteristic on the cranial and cervical elements that can be compared with *Lishulong*: both surangular and angular extended more anterior to the external mandibular fenestra (Wang, You & Wang, 2017). This feature should be attributed to the boundary of the external mandibular fenestra demarcated smaller than in reality, and the displacement of the surangular and angular caused by compression. However, except for the crescent supraoccipital of *Lishulong* mentioned above, the anterolateral expansion on the dorsal surface of the cervical neural spine is absent in all three specimens of *Xingxiulong*. The two taxa are hence clearly different from each other.

The latest-diverging sauropodiforms currently known from the Lufeng Formation, *i.e. Yizhousaurus sunae* is easily characterized by its unique cranial structures, such as the lateral plates of the upper jaws, the shrunken antorbital and external mandibular fenestrae and the vertical lacrimal (Zhang et al., 2018). The intercentrum of the axis of *Yizhousaurus* is wider than its centrum; however, it is narrower than that of *Lishulong*. Besides, the cervical neural spines of *Yizhousaurus* have a constant width throughout its length without the anterolateral expansion as well.

*Jingshanosaurus* and *Yunnanosaurus* have the closest relationships with *Lishulong* displayed in the strict consensus tree (Fig. 6). Although the holotype of *Jingshanosaurus xinwaensis* (LFGT-ZLJ0113) does not have enough preserved cervical vertebrae for comparison, the cranium of its referred specimen (CXM-LT9401) have been reexamined by Zhang et al. (2020) with amended cranial diagnoses for *Jingshanosaurus*. The differences between the two genera are mainly concentrated in tooth morphology: the teeth of *Jingshanosaurus* are imbricated arranged with tooth crowns distally recurved, whereas the teeth of *Lishulong* are linearly placed without any overlapping and with slightly lingual concavities. The dorsal process of the premaxilla of *Jingshanosaurus* has an inflection at the base, but *Lishulong* has a slightly convex premaxillary nasal process. The posterior margin of the external naris of *Jingshanosaurus* is more posterior to the midlength of its maxillary tooth row, which is one of the emended autapomorphies of this genus (Zhang et al., 2020), while its level of *Lishulong* is just posterior to the first maxillary alveolus, as in most non-sauropodan sauropodomorphs. Furthermore, the position of the jaw joint in *Jingshanosaurus* is well below the level of the dorsal margin of the dentary, whereas that of *Lishulong* is flush with the dentary dorsal margin. The supraoccipital is higher diamond-shaped in *Jingshanosaurus*, as in most non-sauropodan sauropodomorphs, unlike the lower crescent-shaped of *Lishulong*.

*Yunnanosaurus huangi* is also reported by Young (1942) and redescribed by Barrett et al. (2007), which has been included in several cladistic analyses with different attribution among sauropodomorphs (Martínez & Alcober, 2009; McPhee et al., 2015; Otero & Pol, 2013; Sereno, 1999; Upchurch, Barrett & Galton, 2007). These bifurcations could be caused by the poor preservation of the cranium of *Yunnanosaurus* and its holotype could belong to a subadult individual (Q-N Zhang and H-L You, 2018, personal observation). The amended cranial diagnoses of *Yunnanosaurus* are similar to those of *Lufengosaurus* mentioned above, such as the ventral projection of the maxillary ascending process, the midline boss near the frontal, and the sub-circular fossa on the lacrimal ventral process are all caused by the preservation conditions; the lack of nutritive foramina, and the tooth denticles of the maxilla perhaps because of scraped bone surfaces or the matrix coverage. Nonetheless, the subtriangular cranial shape, the anteroposteriorly expanded premaxillary nasal process, and the small external naris of *Yunnanosaurus*, distinguish it from *Lishulong*.

It is clear, therefore, that the validity and interrelationships of early sauropodiforms are still to be determined. Our reassessment of the above Lufeng sauropodomorphs has shown that *Lishulong* should be erected as a new taxon. Perhaps both *Yunnanosaurus* and *Lishulong* will be grouped into ‘Jingshanosauridae’ in the future after the ontogenetic information of *Yunnanosaurus* is supplemented. Further assessment of these cladistic issues also requires some foundational work, including detailed research on the post-cranial materials of *Lufengosaurus* and *Jingshanosaurus* that are currently being studied, and separate scoring of the characteristics of the different three individuals of *Xingxiulong*.

### Paleobiogeographic implications

*Lishulong wangi* represents a new member of the seemingly ever-growing diversity of early sauropodiforms in the Lufeng dinosaur fauna. The accumulation of morphological data and phylogeny reconstruction results for non-sauropodan sauropodiforms allow us to conduct a quantitative analysis on the origin center and dispersal routes of this group. Furthermore, they possibly provide some new insights into paleobiogeographic mechanisms driving the early diversification of these dominant terrestrial herbivores, which have been appreciated only in relatively recent years.

Despite having an extensive distribution and a relatively long period of exploration of the Late Triassic continental strata in China (*e.g*., Yanchang Formation in the Ordos basin and Xujiahe Formation in the Sichuan basin, Tong et al., 2019), no body fossils of dinosaurs have been hitherto discovered during this geological period, other than two localities from the Xujiahe Formation in the Sichuan basin have been reported to yield trace fossils that possibly originated from theropods (Xing et al., 2013a; Ye, Jiang & Peng, 2016), and two other footprint records that are uncertain about whether they belong to sauropodomorphs (Xing et al., 2013b; Xing et al., 2018). Within the entire East and Southeast Asia, the only dinosaur genera of purported Late Triassic age is an early sauropod (though our phylogenetic analysis has recovered it as an immediate sister-taxon to the node Sauropoda), *i.e*., *Isanosaurus attavipachi* from the Nam Phong Formation of northeast Thailand (Buffetaut et al., 2000). However, subsequent palynological studies indicated that the dating of the Nam Phong Formation was problematic and the horizon from which the sauropod materials had been excavated was most likely of Jurassic age (Laojumpon et al., 2017; Racey & Goodall, 2009). The conspicuous lack of substantial evidence of dinosaurs around East and Southeast Asia is not necessarily evidence of absence; however, if we assume that dinosaurs, or at least sauropodomorphs and ornithischians, had not spread as far east as this geographical area until the Early Jurassic epoch, then the Lufeng Saurischian Fauna dominated by diverse non- sauropodan sauropodomorphs from Lufeng Formation in Yunnan represents the earliest biogeographic occurrence of dinosaurs in this region. This scenario, partially contradicts traditional notions about early dinosaur distribution in the Late Triassic epoch that major dinosaur lineages were assumed to have been cosmopolitan and homogeneous, and ease of dispersal across Pangaea should have lowered the diversity of early dinosaurs (Sereno, 1997, 1999, 2007). However, in the case of East and Southeast Asia, until the end of the Triassic Period, at least some clades of dinosaurs (Sauropodomorpha and Ornithischia) had not spread into these regions. As Marsh & Rowe (2018) have already discussed, the superficial homogeneity of these early dinosaur faunas merely reflects a poor taxonomic resolution, which has been greatly improved through a systematic apomorphy-based method (Nesbitt, 2011). McPhee & Choiniere (2017) also recently summarized that the Early Jurassic sauropodomorphs from the upper Elliot Formation are more diverse taxonomically than we originally believed.

Our phylogenetic analysis has not recovered an endemic clade constituted solely by taxa from Lufeng Formation, *i.e*. they do not form a monophyletic group. This interrelationship of these dinosaurs seems to repeat patterns seen in North American counterparts (Marsh & Rowe, 2018), indicative of multiple dispersal events from different parts of Pangaea. The comparisons of the six biogeographic models in our biogeographic analyses adopting the ‘relaxed’ dispersal multiplier matrix indicate that the + J versions are significantly better fit to the data than the non + J versions, with *p*-values ranging from 1.3e^−13^ to 1.3e^−9^. The AIC value for the BAYAREALIKE + J model is the lowest among the six biogeographic models. The AIC value for the DEC + J model is only slightly higher than that of the BAYAREALIKE + J model (3.7 units higher than the latter). These results demonstrate that the BAYAREALIKE + J model has the best performance in producing the available data, followed by the DEC + J model (Table S3). Therefore, the biogeographic history of these early-diverging sauropodomorphs in our analyses is best explained by sympatry, *i.e*., the direct inheritance of early ancestors’ range (because the BAYAREALIKE + J model only allows range duplication when cladogenesis occurs), as well as regional extinction and founder-event speciation (Landis et al., 2013). The role of vicariance is supported by the second best-performing model, which indicates that vicariance is not as important a driving mechanism as originally believed at that time (Sereno, 1997, 1999, 2007). The taxa in our analyses include some relatively late sauropod lineages (*e.g*., *Mamenchisaurus, Cetiosaurus etc*.), which may explain why the AIC value for DEC + J model is not significantly higher. As discussed by Marsh & Rowe (2018), various factors such as competition, vicariance, extinction, and dispersal do not act uniformly spatiotemporally; therefore, the addition of late sauropods may affect the ultimate AIC assessment for each biogeographic model. The results of analyses adopting the ‘harsh’ dispersal multiplier matrix are quite similar to the aforementioned ones (Table S3).

**Figure 7:**
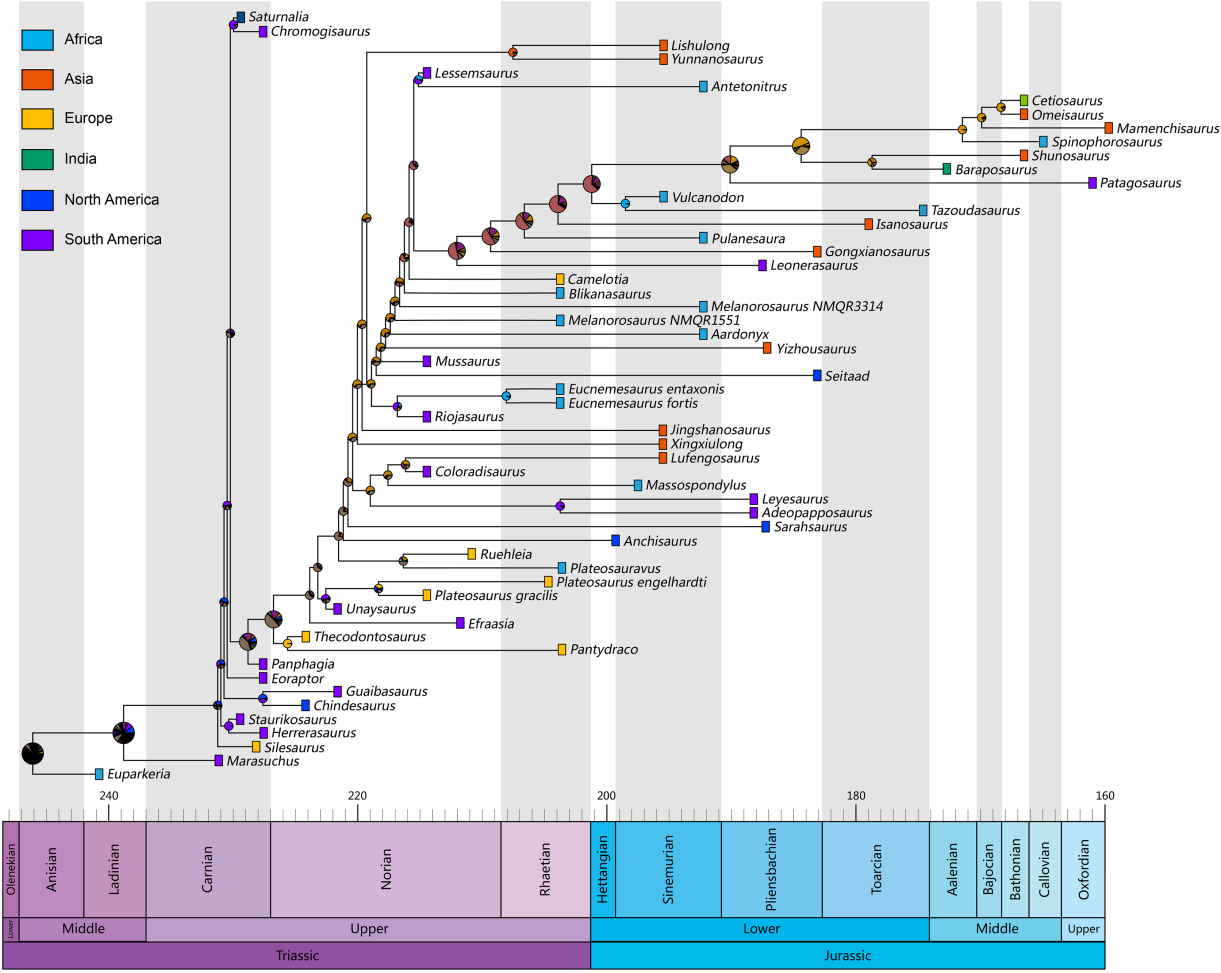
Time-calibrated evolutionary tree for non-sauropodan sauropodomorphs. The ancestral area estimation results of the BioGeoBEARS analysis based on the BAYAREALIKE + J model which best fits the data. The pie charts at each node represent the relative probabilities of the estimated ancestral states, the color of the pie chart represents different ancestral ranges estimations. The time-calibrated tree is produced by the R package ‘trap’ using the ‘equal’ parameter. The timeline below is based on v 2020/03 of the International Chronostratigrahic Chart (http://www.stratigraphy.org/).

Although multiple dispersal events are indicated in the topology of our phylogenetic cladogram, the results of ancestral area reconstruction for Lufeng sauropodomorphs are quite ambiguous (Fig. 7; Table S4). Resembling the results of biogeographic analyses using BioGeoBEARS packages in Poropat et al. (2016) and Xu et al. (2018), some seemingly anomalous discrete area reconstructions are also made in our results. Similar interpretations in the above two studies that link these areas are absent because of sampling failures that could also apply to our analyses. More future finds in these areas and phylogenetic results with higher resolutions may elucidate the exact place of origin of these sauropodomorph taxa. Notably, the ancestral area reconstruction result for the node Sauropodomorpha is South America, a quantitative result similar to those of the recent researches (Marsola et al., 2019; Lee et al., 2018).

## Conclusions

The anatomy of the early-diverging sauropodomorphs from Lufeng is enhanced by the discovery of new material and redescription of previously found material in light of newer material. Through morphological comparisons combined with the phylogenetic analyses, we showed that the specimen of *Lishulong wangi* cannot be classified into any previously discovered genera. As an Early Jurassic form, *Lishulong* increases the growing number of non-sauropodan sauropodomorphs worldwide, among which eight genera are currently known (in addition to the six genera included in our dataset, ‘*Gyposaurus*’ and *Xixiposaurus* are the other two taxa under re-examination for future studies) from the Lower Jurassic series of the same region in China. Our reassessment of the early sauropodiforms closely related to *Lishulong* highlights additional information that can be obtained from an in-depth re-examination of historically collected and poorly characterized Chinese taxa. Further fossil sampling, as well as the comprehensive reanalysis of other poorly known taxa (*e.g*., *Yimensaurus*, *Chinshakiangosaurus* and *Kunmingosaurus*) will be necessary to corroborate the above observations and to better elucidate the contribution of the Chinese Early Jurassic fossil records to our understanding of the general sauropodomorph evolution.

Notably, *Lishulong* has the largest skull among the abundant sauropodomorph members from Lufeng; therefore, it provides a reconsideration for phylogenetic analyses using individual specimens of ascertainable ontogenetic stages as operational taxonomic units to obtain better resolution in general. Our research has provided new insights into previous authors dealing with the anatomy of those Lufeng taxa, representing the first step towards a re-evaluation of this famous dinosaur fauna. Moreover, the paleobiodiversity of early sauropodomorphs from Gondwana seems to decrease marginally after the Triassic-Jurassic boundary. Therefore, we hypothesize that non-sauropodan sauropodomorph genera survived and rapidly radiated in Laurasia, especially China. Furthermore, the ancestral area reconstruction for Lufeng sauropodomorphs is temporarily ambiguous; however, the results of multiple dispersions and exchanges can explain the continuing diversification superiority of non-sauropodan sauropodomorphs from the LJLF. The limited paleobiogeographic information available from *Lishulong* provides evidence that at least the initial sauropodiform lineages that are closely related to near-Sauropoda or Sauropoda existed in southwestern China during the Early Jurassic epoch.

## Supplemental Information

10.7717/peerj.18629/supp-1 Supplemental Information 1 Supplemental Materials.

10.7717/peerj.18629/supp-2 Supplemental Information 2 The phylogenetic analyses in this article based on the modified matrices by Zhang et al. (2018, 2020).

10.7717/peerj.18629/supp-3 Supplemental Information 3 BioGeoBEARS Scripts.

## Institutional abbreviations

CXM: Chuxiong Prefectural Museum, Chuxiong, China
IVPP: Institute of Vertebrate Paleontology and Paleoanthropology, Chinese Academy of Sciences, Beijing, China
LFGT: Bureau of Natural Resources of Lufeng County, Chuxiong, China
NJGM: Nanjing Geological Museum, Nanjing, China
NM: National Museum, Bloemfontein, South Africa

## Additional Information and Declarations

### Data Availability

The following information was supplied regarding data availability: The raw data is in the Supplemental Files.

### New Species Registration

The following information was supplied regarding the registration of a newly described species: Genus name: urn:lsid:zoobank.org:act:EE7FDF02-D7E8-422C-9853-62D7AD5271B2 Species name: urn:lsid:zoobank.org:act:A1B589CE-0836-4048-B68E-3608F59FE574 Publication LSID: urn:lsid:zoobank.org:pub:6A0C59EE-614C-462D-94C6-C28F86B3F9E7.
