# Supplementary material for: The largest sauropodomorph skull from the Lower Jurassic Lufeng Formation of China
Source: PeerJ. 2024 Dec 12;12:e18629. doi: 10.7717/peerj.18629 (PMC11646418; doi:10.7717/peerj.18629)
Supplement: Supplemental Information 1 [file peerj-12-18629-s001.docx]

**SUPPLEMENTARY INFORMATION**

**The largest sauropodomorph skull from the Lower Jurassic Lufeng Formation of China**

Qian-Nan Zhang^1, 2*^, Lei Jia^1, 3, 4^, Tao Wang^5^, Yu-Guang Zhang^6^, Hai-Lu You^1, 3*^

^1^ Key Laboratory of Vertebrate Evolution and Human Origins, Institute of Vertebrate Paleontology and Paleoanthropology, CAS, Beijing, China

^2^ State Key Laboratory of Palaeobiology and Stratigraphy, Nanjing Institute of Geology and Palaeontology, CAS, Nanjing, China

^3^ University of Chinese Academy of Sciences, Beijing, China

^4^ ShanXi Museum of Geology, Taiyuan, China

^5^ Dinosaur Fossil Conservation and Research Center, Bureau of Natural Resources of Lufeng County, Chuxiong, China

^6^ National Natural History Museum of China, Beijing, China

Corresponding authors*: Qian-Nan Zhang and Hai-Lu You

Email address: [zhangqiannan@ivpp.ac.cn](mailto:zhangqiannan@ivpp.ac.cn) or youhailu@ivpp.ac.cn

1. Character states of *Lishulong wangi* used in the phylogenetic analysis in current study.

| ?0011002?0 | 1021111110 | ?01?1010?? | 010000?101 | 11?0?????? |
| --- | --- | --- | --- | --- |
| ??01?1???? | ???????10? | 100?1010?? | 20????0?1? | ???0001101 |
| 0100110100 | 0101011101 | ??00111100 | 1000110101 | ?0110???0? |
| ?????????? | ?????????? | ?????????? | ?????????? | ?????????? |
| ?????????? | ?????????? | ?????????? | ?????????? | ?????????? |
| ?????????? | ?????????? | ?????????? | ?????????? | ?????????? |
| ?????????? | ?????????? | ?????????? | ?????????? | ?????????? |
| ?????????? | ???? |  |  |  |

2. **Supplementary Figure 1.** Photographs of the cranium of *Lishulong wang*i in posterior (A) and ventral (B) views. Abbreviations: so, supraoccipital.

**
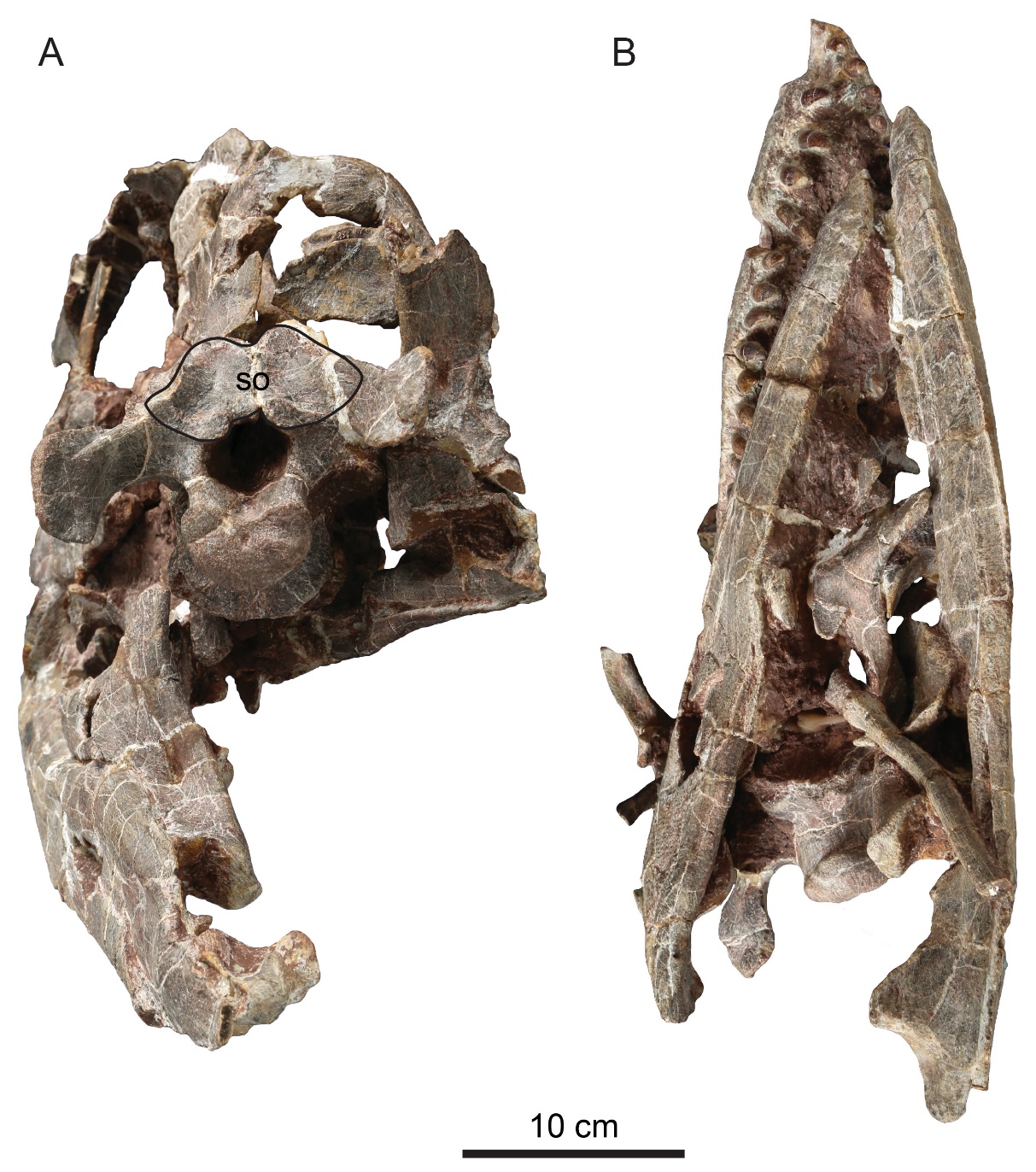
**

3. **Supplementary Figure 2.** Close-up photograph of the tooth row of *Lishulong wang*i in left view.


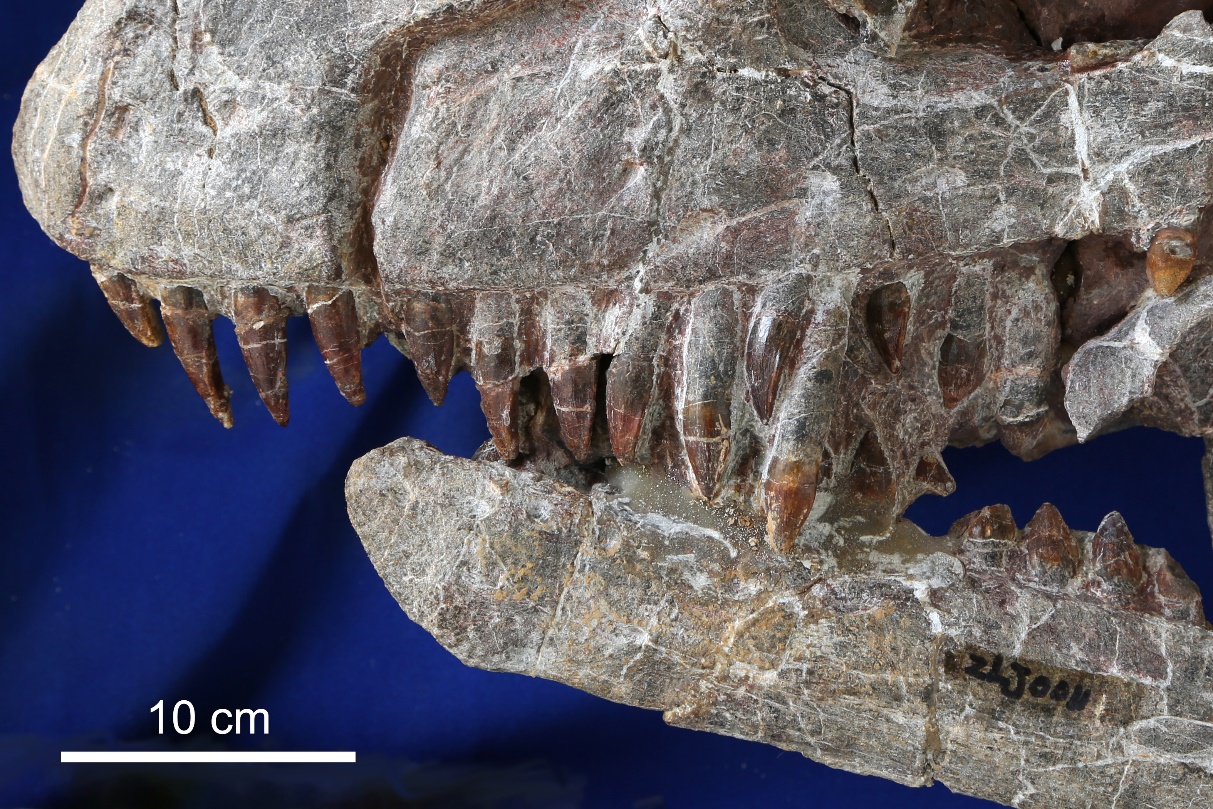


4. **Supplementary Table 1.** measurements (unit: cm) of the cervicals of *Lishulong wangi*. Abbreviations: AnF, anterior face of the centrum; C, centrum; Ca, caudal vertebrae; Ce, cervical vertebrae; D, dorsal vertebrae; Di, diapophysis (mediolateral length); Hy, hyposphene; Mc, metacarpals; NA, neural arch; NS, neural spine (height: measured from the dorsal margin of the postzygapophysis to the top of the neural spine; length: measured at midspine height anteroposteriorly); Pl, phalanges; PoF, posterior face of the centrum; PoZ, postzygapophyses; PrZ, prezygapophyses; S, sacral vertebrae; TP, transverse processes (mediolateral length). * represents estimated value.

| Element | Height | C length | C  Height | AnF  Height | AnF  Width | PoF  Height | PoF  Width | NS  Height | NS  Length | NS  width (top) | PrZ  Length | PrZ  Width | PoZ  Length | PoZ  Width |
| --- | --- | --- | --- | --- | --- | --- | --- | --- | --- | --- | --- | --- | --- | --- |
| Axis | 10.5 | 16.3 | 5 |  | 5 | 4.8 | 5.2 | 2.4 |  | 0.5 |  |  | 7.8 | 3.4 |
| Ce3 | 9.9 | 22.9 | 5 | 5.1 | 6.5 | 5.8 | 6 | 2 | 10.1 | 0.7 | 11.3 | 3.3 | 7.5 | 3.8 |
| Ce4 | 12.9 | 25.6 | 6.5 | 6.5 | 6.8 | 7.5 | 7 | 3 | 10.9 | 1.3 | 13.1 | 3.5 | 8 | 4.8 |
| Ce5 | 15.2 | 26.3 | 7.2 | 8 | 7.8 | 8.5 | 8.5 | 4.1 | 11 | 1.5 | 14.7 | 4.5 | 8.7 | 5.5 |
| Ce6 | 17.3 | 27.3 | 8.3 | 8.7 | 8.8 | 9.9 | 11.4 | 4.5 | 11 | 1.7* | 15.6 | 5 | 8.9 | 6.6 |
| Ce7 | 19.1 | 27.2 | 9.8 | 9.8 | 9.8* | 9.9 | 15 | 5.7 | 11.4 | 1.8 | 15.7 | 5.6 | 9.7 | 6.8 |
| Ce8 | 21.6 | 25.2 | 11.9 | 11.5 | 10.4 | 13.2 | 16 | 5.8 | 10.4 | 2.0 | 14.2 | 6.2 | 9 | 6.5 |
| Ce9 | 25.0 | 23.0 | 11.7 | 12.8 | 12.4 | 15.1 | 17.8 | 6.2 | 8.2 | 4.9 | 12.6 | 6.1 | 10.1 | 6.5 |
| Ce10 | 27.2 | 19.9 | 10.1 | 15.0 | 15.1 | 16.6 | 18.8 | 8.4 | 7.0 | 6.9 | 11.8 | 6.8 | 9.3 | 6.6 |

5. **Supplementary Table 2.** Statistics and sources of measurements (unit: cm) related to Figure 7. * represents estimated value.

| Genus | *Platesaurus* | *Adeopapposaurus* | *Leyesaurus* | *Massospondylus* | *Lufengosaurus* | *Xingxiulong* | *Yunnanosaurus* | *Riojasaurus* | *Yizhousaurus* | *Lishulong* |
| --- | --- | --- | --- | --- | --- | --- | --- | --- | --- | --- |
| Specimen | SMNS 13200 | PVSJ 610 | PVSJ 706 | BP/1/4934 | IVPP V15 | LFGT-D0003 | IVPP V20 | ULR 56 | LFGT-ZLJ0033 | LFGT-ZLJ0011 |
| Skull length | 34.2 | 13.9* | 14.7 | 23.2* | 25.0* | 23.5* | 20.5 | 24.7* | 28.0* | 40.0 |
| Cervical length | 13.0 | 6.8* | 9.7 | 15.2* | 12.8 | 13.5 | 10.5 | 9.2* | 22.1 | 27.3 |
| Cervical No. | 8 | 7 | 7 | 4 | 6 | 5 | 4 | 5 | 7 | 6 |
| Reference | Huene 1926 | Martínez 2009 | Apaldetti et al. 2011 | Barrett et al. 2019 | Young 1941 | Wang et al. 2017 | Young 1942 | Bonaparte & Pumares 1995 | Zhang et al. 2018 |  |

6. **Supplementary Table 3.** Summary of the statistical comparisons between the six biogeographic models adopted in the BioGeoBEARS package. The model marked with an asterisk (*) is the one which best fits the available data. ‘Harsh’ and ‘Relaxed’ in the table refer to the two different dispersal multiplier matrices that were adopted in the two analyses.

| **Analysis** | **Model** | **LnL** | **Log likelihood ratio test** | | **AIC Analysis** | | |
| --- | --- | --- | --- | --- | --- | --- | --- |
|  |  |  | **D statistic** | **P value** | **AIC** | **AICwt** | **Ratio** |
| Relaxed | DEC | -200 |  |  | 404 | 3.90E-09 |  |
|  | DEC+J | -179.7 | 40.71 | 1.80E-10 | 365.3 | 1 | 2.55E+08 |
|  | DIVALIKE | -224.9 |  |  | 453.9 | 3.40E-12 |  |
|  | DIVALIKE+J | -197.5 | 54.81 | 1.30E-13 | 401.1 | 1 | 2.93E+11 |
|  | BAYAREALIKE | -196.2 |  |  | 396.4 | 2.70E-08 |  |
|  | *BAYAREALIKE+J | -177.8 | 36.84 | 1.30E-09 | 361.6 | 1 | 3.67E+07 |
| Harsh | DEC | -200 |  |  | 404 | 6.70E-09 |  |
|  | DEC+J | -180.2 | 39.66 | 3.00E-10 | 366.3 | 1 | 1.50E+08 |
|  | DIVALIKE | -234.4 |  |  | 472.9 | 1.90E-15 |  |
|  | DIVALIKE+J | -199.5 | 69.77 | 6.70E-17 | 405.1 | 1 | 5.20E+14 |
|  | BAYAREALIKE | -196.2 |  |  | 396.4 | 2.50E-08 |  |
|  | *BAYAREALIKE+J | -177.7 | 37 | 1.20E-09 | 361.4 | 1 | 3.99E+07 |

7. **Supplementary Table 4.** Ancestral range reconstruction results for sauropodomorphs from Lufeng Formation, based on the results of the BAYAREALIKE+J Model which best fits the data. ‘Harsh’ and ‘Relaxed’ are as explained in the Table 3. ‘First corner’ and ‘First node’ are in the sense of the definition in Poropat et al. 2016.

|  | ***Lufengosaurus*** | | ***Xingxiulong*** | | ***Jingshanosaurus*** | | ***Lishulong + Yunnanosaurus*** | | ***Yizhousaurus*** | |
| --- | --- | --- | --- | --- | --- | --- | --- | --- | --- | --- |
| **Analysis** | **First corner** | **First node** | **First corner** | **First node** | **First corner** | **First node** | **First corner** | **First node** | **First corner** | **First node** |
| Harsh  BAYAREALIKE+J | Asia+South America | Asia+Africa+North America | Asia+Africa+North America | Asia+Africa+North America | Asia+Africa+North America | Asia+Africa+North America | Asia+Africa+North America | Asia+Africa+North America | Asia+Africa+North America | Asia+Africa+North America |
| Relaxed  BAYAREALIKE+J | Asia+South America | Asia+Africa+North America | Asia+Africa+North America | Asia+Africa+North America | Asia+Africa+North America | Asia+Africa+North America | Asia+Africa+North America | Asia+Africa+North America | Asia+Africa | Asia+Africa+North America |
